# Lost Letter Measure of Variation in Altruistic Behaviour in 20 Neighbourhoods

**DOI:** 10.1371/journal.pone.0043294

**Published:** 2012-08-15

**Authors:** Jo Holland, Antonio S. Silva, Ruth Mace

**Affiliations:** Department of Anthropology, University College London, London, United Kingdom; University of Zaragoza, Spain

## Abstract

Altruistic behaviour varies across human populations and this variation is likely to be partly explained by variation in the ecological context of the populations. We hypothesise that area level socio-economic characteristics will determine the levels of altruism found in individuals living in an area and we use a lost letter experiment to measure altruism across 20 neighbourhoods with a wide range of income deprivation scores in London, UK. The results show a strong negative effect of neighbourhood income deprivation on altruistic behaviour, with letters dropped in the poorest neighbourhoods having 91% lower odds of being returned than letters dropped in the wealthiest neighbourhoods. We suggest that measures of altruism are strongly context dependant.

## Introduction

Altruistic behaviour toward unrelated individuals is likely to depend on future opportunities for reciprocation, reputation enhancement or benefits to the cultural group that benefit the individual indirectly [1]. The level of altruism observed in a population is likely to vary according to its ecological context; for example those with stable lifestyles may have longer time horizons and thus weight future benefits against immediate ones, and those in poverty may prefer quick rewards even if there is a risk of incurring punishment or loss [2]; those with more close kin in the population around them may be more altruistic [3] and those in competition with other groups may be less altruistic to the out-group and more altruistic to the in-group [4].

The lost letter experiments were one of the original experiments that showed altruistic behaviour towards complete strangers, in situations unlikely to elicit any reward, in which a substantial number of city dwellers were found to post back lost stamped letters left on the pavement [5], [6]. Evolutionary economists and anthropologists in particular, have been using a range of economic games and a few ‘real life’ measures in an attempt to quantify the variation in altruistic behaviour between individuals and between populations [7]. Results have so far been mixed, suggesting that the precise details of the ecological context mediating altruism are important and their effect is not yet fully understood. Alexander & Christia [8] found that ethnic differences in Mostar, Bosnia can enhance co-operation in some institutional contexts but not others. In an urban context, Wilson et al. [9] found that the quality of a neighbourhood (estimated from self-reported levels of support from family, school and neighbourhood) in Binghampton, U.S.A. positively predicted the number of dropped letters that were picked up and posted back. Nettle et al. [10] also found that a poor area of Newcastle, UK showed fewer incidences of returning a lost letter, giving to charity in the context of an economic game and generally less health seeking behaviour, when compared with a rich area of Newcastle – but with only two points of comparison it is not possible to determine what aspects of the varying socio-economic conditions underlie these differences. However in contrast, Piff et al. [11] found that wealthy individuals were less likely to behave altruistically than less wealthy individuals in a range of measures, mainly measured from students at University of California at Berkeley, USA.

The lost letter method remains one of the best ways of measuring truly altruistic behaviour that is likely to result in negligible benefits, and incurs a small cost on the person posting the found letter. Here we used a lost letter technique [5], [6] to measure the levels of altruism in neighbourhoods in London with a range of different socio-economic characteristics. We predicted that individuals in more affluent neighbourhoods would behave in a more altruistic manner than individuals in less affluent neighbourhoods. Using the number of returned letters as a proxy for altruistic behaviour, we expected that letters dropped in more affluent areas were more likely to be returned. We also investigated how other neighbourhood characteristics may help explain the variation in altruistic behaviour. We used the percentage of individuals that are UK born as a proxy of ethnic diversity to test whether ethnically mixed neighbourhoods are less altruistic than more ethnically homogeneous ones, and we used distance travelled to work as a proxy for social cohesion, making the assumption that neighbourhoods where people live and work in the same area may result in higher levels of social cohesion. We also used the average house value of the street where the letter was dropped to obtain a more fine-grained measure of wealth. We controlled for population density and the number of post boxes in the neighbourhood.

## Methods

### Sampling Procedure

We dropped 300 letters in 20 neighbourhoods (15 letters per area) in London during June 2010. The letters were addressed by hand to the author's home address with a neutral name (J. Holland), which could have been male or female. The letters were dropped on the pavement with the address face up during rain free weekdays. The neighbourhoods used in this experiment were selected to include a wide range of wealth based on their levels of income deprivation, as measured by a composite index of the proportion of the population experiencing deprivation related to low income [12]. Each neighbourhood represented a Lower Super Output Area (LSOA) which has an average population of 1500. LSOAs are suitable because they are the smallest division where aggregate socio-economic data can be gathered [13].

### Analysis

The outcome variable was a binary variable indicating whether a letter was returned (61%) or not (39%). We regressed this outcome against several predictors: i) income deprivation score (ordinal variable; 4 quartiles; the higher the score the more deprived [12]); ii) population density (continuous variable); iii) percentage of individuals born in the UK (continuous variable); iv) number of post boxes (continuous variable); v) average distance to workplace (continuous variable); vi) house value (continuous variable; 8 bands of average house price for each street [14]); In a preliminary analysis there was an indication of a non-linear effect of the income deprivation score and as result this variable was transformed into an ordinal measure, divided by 4 quartiles. Measures i to v are neighbourhood level variables, while house value is related to the street and hence is a letter level variable. We had also planned on using neighbourhood crime scores as a predictor variable to attempt to disentangle the effects of income deprivation and crime on levels of altruism, but due to strong collinearity (r = 0.90) between these two variables only income deprivation was used in the final model.

We used a logistic random-intercept regression model to measure the amount of variation in returned letters explained by our predictor variables, using the neighbourhood LSOA code as a random effect to take into account the amount of variation that is due to the cluster effects of each neighbourhood [15]. Initially, we ran a null model with no predictor variables to calculate the amount of variation due to between neighbourhood differences, followed by a model with all relevant predictor variables to calculate the amount of this variation explained by the variables used in this model. The models were run using xtlogit in Stata 11.2 with a maximum likelihood estimation procedure.

## Results

**Figure 1:**
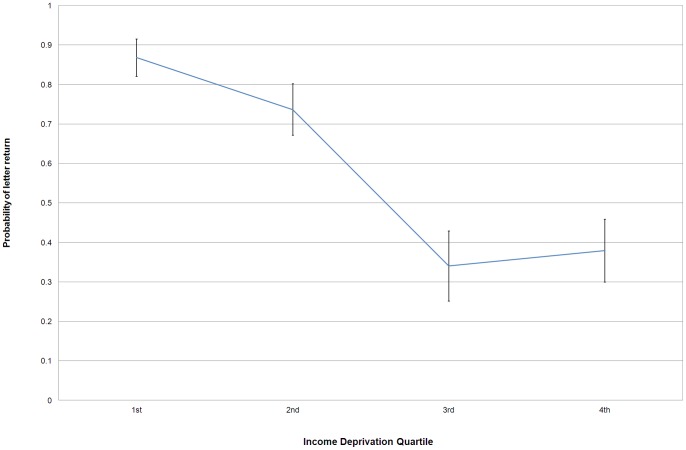
Plot of the predicted probability of a letter being returned by the level of neighbourhood income deprivation score. This measure is a composite index of the proportion of the population experiencing deprivation related to low income and has been divided into 4 quartiles, with the higher the score the more deprived the neighbourhood. This relationship is controlled for house values on the street where the letter was dropped, population density, percentage of UK born individuals, number of post boxes and average distance travelled to work in the neighbourhood. Error bars represent 95% confidence intervals.

We found income deprivation to be the best predictor of whether a letter would be returned or not. On average, 87% of the letters dropped in the richest neighbourhoods (1^st^ quartile) were returned when compared to a 37% return rate in the poorest neighbourhoods (4^th^ quartile) (Table S1). A letter dropped in poorer neighbourhoods (3^rd^ and 4^th^ quartile of income deprivation) had, respectively, 92% and 91% less odds of being delivered than letters dropped in the richest neighbourhood (1^st^ quartile). There was a statistically significant difference between the top 2 quartiles and bottom 2 quartiles (unpublished results). The effect of income deprivation is not linear, with the probability of a letter being returned only increasing on wealthier neighbourhoods with income deprivation scores below 0.16 (Figure 1). Density of post-boxes was controlled for and not found to be a significant predictor of returns. With the exception of average distance travelled to work, which had a positive effect, the other predictor variables were found to have no significant impact in explaining the variance in the return of lost letters (Table 1).

**Table 1:** Descriptive statistics, odds ratio and 95% confidence intervals for the logistic random intercept regression models used to predict letter return rates.

Predictor	Mean (SD)	Odds Ratio	95 CI
Income Deprivation Score			
1Q (0.01–0.02); n = 105 (ref. category)		1.00	-
2Q (0.03–0.16); n = 60		0.43	0.15;1.21
3Q (0.24–0.56); n = 60 ***		0.08	0.02;0.29
4Q (0.59–0.74); n = 75 ***		0.09	0.03;0.32
House Value (£)	91.7 (59.0)	1.00	1.00;1.00
Population Density (pop./hectare)	77.0 (62.2)	1.00	0.99;1.00
UK Born Population (%)	71.3 (13.1)	0.99	0.96;1.03
Number of Post Boxes	4.0 (1.1)	1.03	0.68;1.55
Distance Travelled to Work (km) *	10.6 (2.3)	1.21	1.01;1.45

*** p<.001; *p<0.05.

All the variables used in the full model are, with the exception of house value, neighbourhood level variables. As a result, if these variables accurately explain the outcome variance for the analysis, we would expect the proportion of variance accounted for by neighbourhoods to be substantially reduced when compared to the null model with no predictor variables. This was confirmed by the decrease of the intra-cluster correlation coefficient at the neighbourhood level from 0.28 in the null model to <0.00 in the full model, demonstrating the validity of the model used.

## Discussion

The likelihood of returning a lost letter at no obvious benefit to the individual is strongly predicted by the level of income deprivation in the local area, and appears to not be greatly influenced by other socio-economic descriptors of the neighbourhood. This suggests that those living in poor neighbourhoods are less inclined to behave altruistically toward their neighbours.

These results could be explained by the characteristics of individuals in poor neighbourhoods (e.g. income and education) or due to the area level characteristics of these neighbourhoods (e.g. social cohesion and crime). Low-income individuals may be too preoccupied with meeting individual needs to spend effort (even if rather trivial) on improving an outcome for an unknown neighbour, or may view their residency in poor areas as temporary and therefore choose not to invest in pro-social behaviour. The lower levels of social cohesion and high incidence of crime normally found in poor neighbourhoods [16], [17] are also likely to affect how individuals behave, irrespective of their individual characteristics, by creating feelings of distrust and leading to less altruistic behaviour towards neighbours. The high correlation between income deprivation and crime scores in our sampled neighbourhoods confirms this association, but it also makes it impossible to assert which factor, poverty or crime, actually predicts altruistic behaviour.

We also found the relationship between income deprivation and returned letters to be non-linear, with no significant differences found between the neighbourhoods in the lower income quartiles or between the higher income quartiles. This suggests a threshold effect, in which individuals start behaving altruistically only above a certain income bracket. This appears to start happening in neighbourhoods with a score above 0.16, ranked in the 30% wealthiest neighbourhoods in England [12]. Our other more fine-grained income measure, average house price, did not have any effect on the rate of returned letters and appears to be too local to have any additional influence – it is the socio-economic profile of the neighbourhood, not of the street that matters. The average distance travelled to work was the only other significant measure, albeit in an opposite direction to the predicted effect, with neighbourhoods where people travelled furthest to work being more altruistic. This variable is unlikely to have measured neighbourhood cohesion as initially expected, but it may be associated with more people walking in these commuter areas on the street to a train or bus station and finding the letters.

Our overall findings replicate and expand on previous studies using similar methodology [9], [10] but are in contrast with the findings of Piff et al [11] who find that wealthy individuals in Berkeley, U.S.A. are more likely to not give way to other cars or pedestrians, and are more likely to behave selfishly or unethically in economic games. One possible explanation for these contradictory results is that Piff et al [11] findings are likely due to individual level differences, whilst our findings may stem mainly from contextual neighbourhood effects. Therefore our results may not be in conflict, if good socio-economic conditions in an area lead to increased trust and long-term thinking, even though, within any one neighbourhood, wealthier individuals are less altruistic than poorer people. If this is the case, we would predict that the lost letters in our experiment were more likely to be returned by the poorer individuals in the area, and that the wealthy residents of Berkeley would behave even less altruistic when in a poorer neighbourhoods. These latter hypotheses have yet to be tested. Alternatively, these contradictory results may be highlighting domain specific differences of altruistic behaviour between rich and poor people; for example anti-social behaviours involving competition (such as aggressive driving or cheating in an economic game) may be more common amongst the wealthy, whereas in a non-competitive task (such as returning a lost letter) wealthy individuals behave more altruistically than poor individuals.

In this study, we have shown that individuals living in poor neighbourhoods are less altruistic than individuals living in wealthier neighbourhoods. However, we have not been able to identify the specific neighbourhood characteristic behind this, due to income being strongly correlated with other factors, such as crime. Further research should focus on attempting to disentangle these two factors, possibly by comparing equally deprived neighbourhoods with different levels of crime.

## Supporting Information

Table S1 **Aggregate data by neighbourhood with total number of returned letters (15 letters dropped per neighbourhood), income deprivation scores and quartiles (the higher the more deprived), population density (pop./hectare), number of postboxes, average distance travelled to work (km) and percentage of the population that is UK born.** Neighbourhoods are sorted by income deprivation.
(DOC)
