# Supplementary material for: Lost Letter Measure of Variation in Altruistic Behaviour in 20 Neighbourhoods
Source: PLoS One. 2012 Aug 15;7(8):e43294. doi: 10.1371/journal.pone.0043294 (PMC3419711; doi:10.1371/journal.pone.0043294)
Supplement: Table S1 — Aggregate data by neighbourhood with total number of returned letters (15 letters dropped per neighbourhood), income deprivation scores and quartiles (the higher the more deprived), population density (pop./hectare), number of postboxes, average distance travelled to work (km) and percentage of the population that is UK born. Neighbourhoods are sorted by income deprivation. (DOC) [file pone.0043294.s001.doc]

Table S1

| **Neighbourhood** | **No. of Letters Returned** | **Income Deprivation Score (Quartile)** | **Population Density** | **No. of Postboxes** | **Distance Travelled To Work** | **% of UK Born Population** |
| --- | --- | --- | --- | --- | --- | --- |
| East Sheen | 11 | 0.01 (1) | 20.42 | 5 | 10.35 | 76.87 |
| Bromley 1 | 13 | 0.02 (1) | 30.47 | 5 | 11.6 | 90 |
| Bromley 2 | 13 | 0.02 (1) | 25.39 | 6 | 10.78 | 87.73 |
| Merton | 14 | 0.02 (1) | 25.94 | 4 | 12.55 | 67.49 |
| Pinner | 14 | 0.02 (1) | 33.8 | 4 | 17.65 | 81.55 |
| Sutton West | 12 | 0.02 (1) | 45.68 | 3 | 11.9 | 89.3 |
| Wimbledon | 14 | 0.02 (1) | 149.12 | 3 | 10.77 | 72.83 |
| Sutton Clockhouse | 10 | 0.03 (2) | 41 | 5 | 9.63 | 89.96 |
| Barnet | 12 | 0.16 (2) | 54.24 | 4 | 10.65 | 65.66 |
| East Acton | 10 | 0.16 (2) | 14.98 | 3 | 9.74 | 51.11 |
| Greenwich | 13 | 0.16 (2) | 33.47 | 6 | 13.84 | 91.99 |
| Lavender | 5 | 0.24 (3) | 57.89 | 5 | 8.64 | 75.82 |
| Canning | 6 | 0.46 (3) | 104.55 | 4 | 11.47 | 69.37 |
| Hoxton | 4 | 0.47 (3) | 262.38 | 5 | 7.73 | 58.91 |
| Shadwell 2 | 4 | 0.56 (3) | 165.8 | 3 | 7.84 | 59.39 |
| Shadwell 1 | 6 | 0.59 (4) | 160.8 | 3 | 11.29 | 58.74 |
| Blackwall | 7 | 0.62 (4) | 75.49 | 2 | 9.2 | 56.71 |
| East India | 5 | 0.64 (4) | 65.76 | 3 | 10.66 | 57.31 |
| Limehouse | 3 | 0.7 (4) | 95.34 | 3 | 7.51 | 67.68 |
| St. Dunstans | 7 | 0.74 (4) | 78.03 | 3 | 8.64 | 57.13 |
